# Editorial: Deepening our understanding of the etiology of gaming disorder and gambling disorder

**DOI:** 10.3389/fpsyt.2023.1266531

**Published:** 2023-08-23

**Authors:** Jiang Long, Chang Qi, Natacha Carragher

**Affiliations:** ^1^Shanghai Mental Health Center, Shanghai Jiao Tong University School of Medicine, Shanghai, China; ^2^Department of Internal Medicine, Yale School of Medicine, Yale University, New Haven, CT, United States; ^3^The Centre of Excellence for Research in AIDS, Faculty of Medicine, Universiti Malaya, Kuala Lumpur, Malaysia; ^4^Department of Psychiatry, Zhejiang Provincial People's Hospital, People's Hospital of Hangzhou Medical College, Hangzhou, Zhejiang, China; ^5^National Drug and Alcohol Research Centre, University of New South Wales, Sydney, NSW, Australia

**Keywords:** gaming disorder, gaming addiction, behavioral addiction, gambling disorder, etiology

## Introduction

In 1990, Isaac Marks introduced the construct of “non-chemical addictions” (1). In the intervening decades, the addiction research field endorsed the term behavioral addictions or addictive behaviors and a body of research has accumulated on the concept. A major step forward for the field in terms of formally recognizing addictive behaviors came in 2015 when gambling disorder was reclassified from the “Impulse Control Disorders Not Elsewhere Classified” category into the new “Substance-Related and Addictive Disorders” category in the fifth edition of the Diagnostic and Statistical Manual of Mental Disorders [DSM-5; (2)] and “internet gaming disorder” was included as a condition requiring further research in the appendix of DSM-5. More recently, in 2019, gaming disorder was included in the eleventh revision of the International Classification of Diseases [ICD-11; (3)] for the first time, and both gaming disorder and gambling disorder were classified in the “Disorders due to addictive behaviors” category. Research to date has identified a variety of complex neurobiological and psychosocial factors implicated in the development and maintenance of gaming disorder and gambling disorder. However, many questions remain about etiology.

Against this background, this Research Topic “*Deepening our understanding of the etiology of gaming disorder and gambling disorder*” aimed to compile and disseminate the latest evidence on pathological mechanisms of gaming disorder and gambling disorder. The evidence helps to better characterize correlates of addictive behaviors, to obtain new insights into underlying pathological pathways and to elicit more comprehensive theories, which can deepen our understanding of the etiology of gaming disorder and gambling disorder. Research teams from Australia, China, India, Japan, Turkey, and the United Kingdom contributed to the topic with their innovative findings from various perspectives.

## Gender and gaming disorder

Gender has long been identified as playing an important role in the development and maintenance of disorders due to addictive behaviors. The literature suggests that males are more vulnerable to developing gaming disorder and gambling disorder albeit, increasingly, the traditional gender divide in gaming is narrowing as more females have become involved in playing video games. In addition, symptomatic patterns tend to be distinct between males and females. As Thakur et al. state, the current understanding on gaming and gambling disorders are largely based on data from male populations. Therefore, their study attempted to bridge the existing gap in the literature by exploring psychopathological characteristics of gaming disorder among female adolescents. Their findings add gender-specific insights to the literature and contribute to early screening and prevention of gaming disorder among female adolescents.

Although the etiology of gaming disorder remains a topic of investigation, some treatments are validated and utilized in clinical settings. Tateno et al. investigated the current practice of gaming disorder in clinical settings in Japan, one of the pioneering countries for research and clinical management for gaming disorder. Their findings provide up-to-date guidance into clinical management which is highly needed in the field. Cognitive and emotional dysfunctions are also central features in gaming disorder. Wang S. et al. aimed to explore how response inhibition, impulse control, and emotion regulation are related to symptomatic severity of gaming disorder. A combination of abnormal emotion regulation and response inhibition was identified as a potential marker for identifying high-risk individuals. The findings help deepen our understanding of the role of emotion regulation and cognitive functions in the etiology of gaming disorder.

## Gaming disorder in the COVID-19 context

COVID-19 has attracted attention from researchers globally, including the field of addictive behaviors. In this issue, research teams from Australia and China investigated gaming and internet use in the context of COVID-19 (Kim et al.; Wang Z. et al.). Using a large longitudinal sample, Kim et al. explored the prevalence and risk factors associated with gaming disorder and identified trajectories of gaming disorder during the pandemic in Australia. Their findings suggest a bidirectional relationship between anxiety and gaming disorder symptoms and highlight the importance of addressing the interactions between risk factors (e.g., anxiety) and gaming disorder in treatment approaches. By using a longitudinal design, Wang Z. et al. contributed to this issue by investigating the relationship between positive youth development (PYD) and internet use in the context of COVID-19 in China. It is indeed a timely and valuable study as a prosocial approach that enhances young people's inherent potential, strengths and capabilities and promotes positive outcomes is particularly valuable during times of crisis, such as COVID-19. The research team found that during the COVID-19 pandemic, the qualities of children and adolescents' PYD declined, which makes children and adolescents more vulnerable to internet addiction. The findings from two distinct cultures provide a unique perspective to behavioral additions.

## National review on gaming disorder

Language poses a barrier to dissemination of scientific findings to the wider academic community and general public as literature published in languages other than English is not widely accessible to international scholars. This is particularly the case in gaming disorder where much research has been conducted in Asia and evidence has been overlooked due to language barriers. Therefore, it is encouraging to see that Boz and Dinç examined the literature for gaming addiction studies in Turkish and summarized and translated the findings into English. Their work presents us with a nuanced perspective of gaming-related research in Turkey and offers an opportunity for comparing trends and patterns in Turkey to other countries/jurisdictions.

## Perspectives

There are many unanswered questions across the etiology of gaming and gambling disorders, as these are relatively new conditions compared to more established psychiatric disorders. Contributions made by the teams from various parts of the world in this Research Topic hold promise for further enhancing our understanding of gaming disorder, internet use and gambling disorder as issues of significant public health concern.

## Author contributions

JL: Conceptualization, Project administration, Writing—original draft, Writing—review and editing. CQ: Conceptualization, Project administration, Writing—review and editing. NC: Conceptualization, Writing—review and editing.

## References

[1] Marks I. Behavioural (non-chemical) addictions. Br J Addict. (1990) 85:1389–94. 2285832 10.1111/j.1360-0443.1990.tb01618.x

[2] American Psychiatric Association DSM-5 Task Force. Diagnostic and Statistical Manual of Mental Disorders: DSM-5™. 5th ed. American Psychiatric Publishing (2013). 10.1176/appi.books.9780890425596

[3] World Health Organization. ICD-11. International Classification of Diseases 11th Revision. Geneva: World Health Organization (2019). Available online at: https://icd.who.int/en

